# Inter-rater reliability of point-of-care ultrasound during out-of-hospital cardiac arrest: an ancillary analysis of the observational prospective ACE trial

**DOI:** 10.1016/j.resplu.2025.101191

**Published:** 2025-12-13

**Authors:** Mathilde Papin, Thibaut Markarian, Quentin Le Bastard, Christelle Volteau, Philippe Pes, Philippe Le Conte, François Javaudin

**Affiliations:** a Service des Urgences, Centre Hospitalier Universitaire de Nantes F-44000 Nantes, France; b Faculté de médecine, Nantes Université, F-44000 Nantes, France; c Nantes Université, CHU Nantes, INSERM, Service des Urgences, CIC 1413, Immuno-infectiologie, F-44000 Nantes, France; d Service des Urgences, Hôpitaux Universitaires de Marseille Timone, F-13005 Marseille, France; e UMR 1263 Centre de recherche en CardioVasculaire et Nutrition (C2VN), Aix-Marseille, Université, INSERM, INRAE, F-13005 Marseille, France; f Nantes Université, CHU Nantes, Cibles et médicaments des infections et du cancer, IICiMed, UR 1155, F-44000 Nantes, France; g Nantes Université, CHU Nantes, Direction de la Recherche et de l’innovation, Plateforme de méthodologie et biostatistique, F-44000 Nantes, France

**Keywords:** Point-of-care ultrasound, Echocardiography, Out-of-hospital cardiac arrest, Cardiopulmonary resuscitation

## Abstract

**Background:**

Prognostication in out-of-hospital cardiac arrest (OHCA) remains challenging. While point-of-care ultrasound (POCUS) has demonstrated utility in identifying reversible causes and predicting outcomes, the quality and reliability of echocardiographic assessments in this context remain poorly characterized. This study aimed to evaluate interrater agreement between trained emergency physicians (POCUS operators) and ultrasound experts in assessing cardiac motion on ultrasound during cardiopulmonary resuscitation (CPR).

**Methods:**

This study was an ancillary analysis of the ACE trial, a multicenter, prospective, observational study conducted between November 2018 and January 2023, which included 293 patients. A random sample comprising 20 % of recorded ultrasound cine loops was included in the present analysis. Two independent experts evaluated the presence of visible cardiac motion. Their assessments were compared with those of the POCUS operator using Cohen’s *κ* coefficients.

**Results:**

A total of 52 POCUS cine loops were collected. The median patient age was 69 years. The presumed etiology of OHCA was cardiac in 61.5 % of cases, noncardiac medical in 32.7 %, and traumatic in 5.8 %. Expert 1 excluded 16 loops (31 %) and Expert 2 excluded 9 loops (17 %) because of insufficient image quality. The *κ* coefficients for detection of cardiac motion were 0.26 (95 % CI, −0.05–0.58) and 0.25 (95 % CI, −0.05–0.54) for agreement between operators and experts 1 and 2, respectively. Inter-expert agreement was higher, with a *κ* of 0.75 (95 % CI, 0.51–0.98). The positive predictive value of cardiac standstill for absence of return of spontaneous circulation (ROSC) did not differ significantly between operators and experts (74.3 % vs 65.0 % and 72.4 %; *p* = 0.47 and 0.87, respectively).

**Conclusion:**

Agreement between emergency physicians and experts regarding POCUS image quality and interpretation of cardiac motion during OHCA was limited. However, this discrepancy did not appear to significantly affect the prognostication of ROSC. Further training and standardization of image acquisition and interpretation criteria may improve POCUS reliability in this setting.

**Trial registration:** This paper is an ancillary study of the ACE trial, registered on ClinicalTrials.gov (Identifier: NCT 03494153) on March 29, 2018.

## Introduction

Out-of-hospital cardiac arrest (OHCA) is a common and extensively studied condition; however, prognostication during cardiopulmonary resuscitation (CPR) remains a significant challenge. It has become a central focus of research in the field of cardiac arrest. In the context of OHCA, termination of resuscitation (TOR) rules are being investigated to provide clinical and paraclinical criteria that guide informed decisions on whether to continue or discontinue resuscitation efforts.^1^

Point-of-care ultrasound (POCUS) during CPR appears to be a valuable tool for identifying reversible causes of OHCA and assessing prognostic factors. POCUS should be performed by trained operators with minimal chest compression interruptions.^2^ The absence of visible cardiac motion during resuscitation is recognized as a marker of poor prognosis in both the short and long term.^3^ However, the definition of “cardiac motion” varies across the literature, ranging from any cardiac movement to specifically myocardial or ventricular movement. Furthermore the conditions under which POCUS is performed (in-hospital vs. out-of-hospital), as well as the timing of the assessments, are heterogeneous.^4^ These variations contribute to considerable heterogeneity in the results reported.^5^

The ACE study specifically investigated the performance of early POCUS (<12 min from the initiation of advanced life support, ALS) in an out-of-hospital setting. POCUS were performed on-site by trained emergency physicians to identify prognostic indicators of return of spontaneous circulation (ROSC). One of its objective was to facilitate integration of POCUS findings into decisions regarding the continuation or cessation of ALS. In the study’s multivariate analysis, only POCUS-confirmed cardiac standstill—defined as the absence of any cardiac movement—and an end-tidal CO_2_ (ETCO_2_) value ≤37 mmHg were independently associated with the absence of ROSC. These findings underscore the prognostic significance of these parameters, which should be considered alongside other TOR rules.^6^

A systematic review conducted by ILCOR highlights the value of ultrasound in this setting but emphasizes the need to address inherent biases in its use—particularly inter-operator reliability—to ensure that it can be widely adopted by all practitioners managing cardiac arrest.^5^ To our knowledge, no current data evaluate the quality of these echocardiographic assessments, especially their inter-operator reliability, given the challenging conditions in which they are performed. Therefore, this ancillary study, based on data from the ACE study, aimed to assess the concordance between trained emergency physicians and ultrasound experts in identifying cardiac motion during CPR.

## Methods

### Study settings

This study is a pre-planned ancillary analysis of the ACE trial, a multicenter, prospective, observational study conducted across 8 Mobile Intensive Care Unit (MICUs) from 5 university hospitals and 3 community hospitals in France.^7^ The ACE trial found that early POCUS cardiac standstill during CPR for out-of-hospital cardiac arrest was a reliable predictor of the absence of ROSC. The inclusion period spanned from November 27, 2018 to January 21, 2023 with a one-year interruption in 2020 due to the COVID-19 pandemic.^6^

### Population

Patients enrolled in the ACE trial were adults experiencing OHCA for whom cardiac POCUS was performed by an emergency physician within 12 min of the team's arrival at the patient’s side.^8^

Expert reviewers analyzed a convenience sample of recorded POCUS loops. Two independent ultrasound experts retrospectively assessed the selected loops. This sample corresponds to the loops that were effectively saved and retrieved for expert analysis (a subset could not be reliably preserved and delivered to the experts).

### Echography

The ultrasound was performed by physicians coming from centers that routinely use ultrasound during cardiac arrest, but they were not selected based on their specific ultrasound training or level of expertise. They all received a “refresh” training to refresh and standardize their knowledge. The ultrasound was performed using the usual hand-helded equipment of the teams that included the patients. The experts are emergency physicians with extensive training and expertise in clinical ultrasound.

### Endpoints and outcomes

The primary objective and endpoint were the assessment of inter-rater agreement between operators and experts regarding the presence or absence of cardiac motion during CPR. The definition of cardiac standstill remains non-consensual; therefore, we defined the presence of motion as “the presence of myocardial movements and/or valvular movements”.

The secondary objectives and endpoints were from one hand to evaluate the agreement between the operator and the experts regarding the quality of POCUS loops. Loop quality was rated on a numeric scale ranging from 0 (very poor) to 10 (excellent). From another hand, to estimate and compare the PPV of POCUS cardiac standstill within the first 12 min of advanced life support (ALS) in predicting the absence of ROSC.

### Statistical analysis

To avoid selection bias, baseline characteristics of the study population were described and compared to those of the overall ACE trial cohort. Continuous variables were summarized using means and standard deviations; categorical variables were reported as frequency and percentages. Group comparisons were conducting using Student’s tests, Chi-square or Fisher’s tests as appropriate.

Primary objective was inter-rater agreement on cardiac motion assessed using Kappa coefficients with 95 % confidence intervals for each pairwise comparison (operator vs expert 1, operator vs expert 2 and expert 1 vs expert 2). Secondary objectives were firstly agreement on loop quality between operators and experts evaluated graphically (scatter plot) and quantitatively using the Intraclass Correlation Coefficient (ICC) and its 95 % CI (comparing operator rating to the average of both experts’ ratings). Secondly, the PPV of POCUS cardiac standstill was calculated and compared between operators and experts using Chi-square tests. Sensitivity, specificity and NPV were also estimated, each with their 95 % confidence intervals. The experts were considered as the gold-standard reference.

All statistical analyses were performed using SAS 9.4 (SAS Institute, Cary, NC, USA). Statistical significance was set at a two-sided *p*-value <0.05.

### Ethical considerations

The ACE trial received approval from the appropriate ethics committee (Comité de Protection des Personnes, 2018-A01491-54). In accordance with the recommendations, informed consent was obtained only for surviving patients or their legal representatives.

## Results

A total of 52 loops were selected for this ancillary analysis. Baseline characteristics are detailed in Table 1. Compared to the overall ACE trial cohort, the characteristics of this subgroup were similar.

**Table 1:** Patient's characteristics.

**Variables (mean ± SD or *n*, %)**	**Total of ACE population** **(*n* = 293)**	**Ancillary study population** **(*n* = 52)**
Age, years	66.6 ± 14.6	65.5 ± 15.57
Sex male	222 (75.8 %)	39 (75 %)
Estimated weight, kg	80.0 ± 18.3	75.9 ± 13.37
**Known medical history**		
Coronary artery disease	48 (20.5 %)	9 (23.68 %)
Chronic heart failure	42 (16.9 %)	9 (21.95 %)
Diabetes	45 (17.9 %)	5 (12.2 %)
Hypertension	124 (49.4 %)	20 (47.62 %)
Chronic obstructive pulmonary disease	25 (10.3 %)	4 (10.53 %)
Liver disease	11 (4.6 %)	2 (5.41 %)
Cancer	33 (13.6 %)	7 (18.92 %)
Neurologic disorder	12 (4.7 %)	4 (9.3 %)
Dementia	4 (1.6 %)	0 (0 %)
Psychiatric disorder	18 (7.1 %)	3 (7.14 %)
Chronic renal failure	10 (4.1 %)	1 (2.5 %)
**Activity limitation**		
Good health	136 (46.4 %)	26 (50 %)
Moderate limitation of activity	119 (40.6 %)	19 (36.54 %)
Chronic disease	21 (7.2 %)	3 (5.77 %)
Severe restriction of activity	2 (0.7 %)	0 (0 %)
**Etiology of cardiac arrest**		
Cardiac	206 (70.31 %)	32 (61.54 %)
Noncardiac medical	76 (25.94 %)	17 (32.69 %)
Traumatic	11 (3.75 %)	3 (5.77 %)
Witnessed cardiac arrest	216 (73.7 %)	35 (67.31 %)
Bystander CPR	223 (76.1 %)	39 (75 %)
**Initial cardiac rhythm**		
Asystole	199 (68.2 %)	29 (55.77 %)
Pulseless electrical activity	52 (17.8 %)	13 (25 %)
Ventricular fibrillation	40 (13.7 %)	10 (19.23 %)
Pulseless ventricular tachycardia	1 (0.3 %)	0 (0 %)
No-flow duration, min	8.2 ± 10.7	7.1 ± 9.88
Time between collapse and ALS, min	28.0 ± 15.3	25.77 ± 16.06
Time between ALS and POCUS, min	7.9 ± 2.6	7.73 ± 2.58
**Cardiac rhythm at POCUS time**		
Asystole	187 (63.8 %)	30 (57.69 %)
Pulseless electrical activity	67 (22.9 %)	14 (26.92 %)
Ventricular fibrillation	38 (13.0 %)	8 (15.38 %)
Pulseless ventricular tachycardia	1 (0.3 %)	0 (0 %)
ETCO_2_ at POCUS time, mmHg	29.7 ± 17.0	35.58 ± 18.1
ROSC	73 (24.9 %)	17 (32.69 %)
ECPR	7 (2.4 %)	2 (3.85 %)
**D30 outcome**		
D30 survival	6 (2.0 %)	1 (1.92 %)
CPC 1	5 (1.71 %)	0 (0.0 %)
CPC 2	1 (0.34 %)	1 (1.92 %)
CPC 3	0 (0.0 %)	0 (0.0 %)
CPC 4	0 (0.0 %)	0 (0.0 %)
CPC 5	287 (97.95 %)	51 (98.08 %)
Organ donation	5 (1,7%)	4 (25 %)

Activity limitations: good health = previous good health, no functional limitations; moderate limitation of activity = mild-to-moderate limitation of activity because of a chronic medical problem; chronic disease = chronic disease causing serious but not incapacitating limitation of activity; and severe restriction of activity = severe restriction of activity due to disease, including being bedridden or institutionalized because of illness.

The presumed etiology of cardiac arrest was cardiac in 32 cases (61.5 %), medical noncardiac in 17 cases (32.7 %), and traumatic in 3 cases (5.8 %). In 75 % of cases, CPR was initiated by bystanders. Upon the arrival of the MICU, the initial cardiac rhythm was asystole in over half the cases. ROSC was achieved in 30 % of patients and 30-day survival was limited to 1.9 %.

**Fig. 1:**
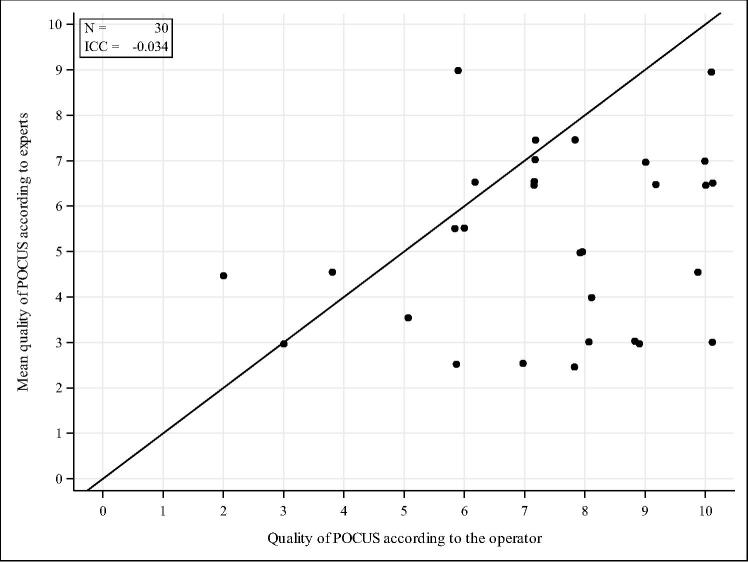
**Comparison of POCUS loops quality (scale from 0 very bad to 10 excellent) according to the experts vs. the operator**.

Two ultrasound experts independently assessed the loop quality. Expert 1 excluded 16 loops (31 %) and Expert 2 excluded 9 loops (17 %) due to insufficient image quality for reliable interpretation of cardiac motion. Overall, operators tended to overestimate loop quality when compared to expert evaluation. The ICC was −0.034, indicating poor agreement (Fig. 1). The median quality of the POCUS was 7 [5.00; 8.00] for the operator and 5 [3.5; 6.5] for the experts.

Among the analysable loops, the agreement for the presence or absence of cardiac motion was evaluated with a kappa coefficient. Between the operator and Expert 1, *κ* = 0.26 (95 % CI [−0.05–0.58]) and between the operator and Expert 2 *κ* = 0.25 (95 % CI [−0.05–0.54]). Between the two experts we found *κ* = 0.75 (95 % CI [0.51–0.98]), as shown in Table 2.

**Table 2:** A = Agreement between expert 1 and operator about presence or absence of cardiac motion. B = Agreement between expert 2 and operator about presence or absence of cardiac motion. C = Agreement between both experts about presence or absence of cardiac motion.

**A**	**Expert 1 = No**	**Expert 1 = Yes**	**Total**
Operator = No	9	6	15
Operator = Yes	7	14	21

Total	16	20	36

**B**	**Expert 2 = No**	**Expert 2 = Yes**	**Total**
Operator = No	8	9	17
Operator = Yes	6	20	26

Total	14	29	43

**C**	**Expert 2 = No**	**Expert 2 = Yes**	**Total**
Expert 1 = No	11	2	13
Expert 1 = Yes	2	18	20

Total	13	20	33

**Fig. 2:**
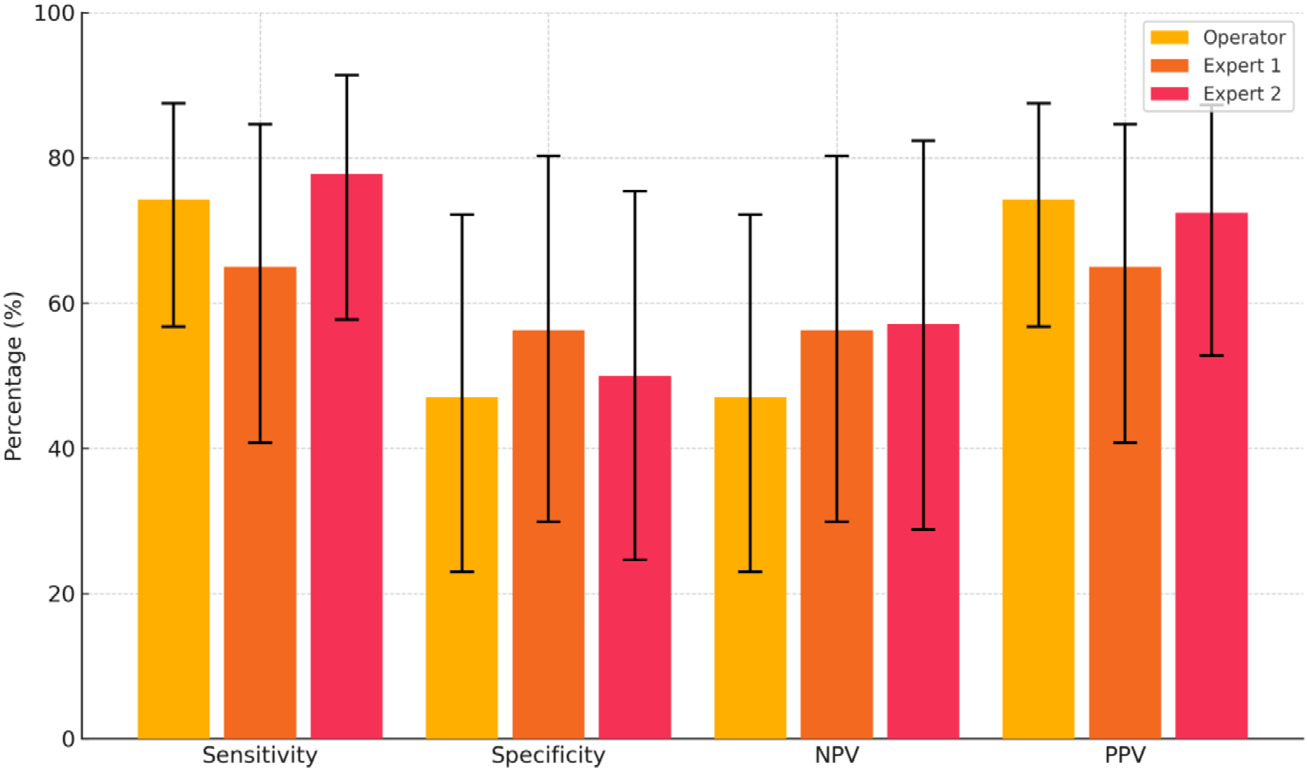
**Diagnostic accuracy according to the operator (yellow), expert 1 (orange) and expert 2 (red) to identify the absence of ROSC**. (For interpretation of the references to color in this figure legend, the reader is referred to the web version of this article.)

The PPV of cardiac standstill, assessed by the operator for predicting absence of ROSC, was not significantly different from those obtained by the two experts: 74.3 % (95 % CI: 56.7–87.5 %) for the operator, versus 65.0 % (95 % CI: 40.8–84.6 %) and 72.4 % (95 % CI: 52.8–87.3 %) for Expert 1 and Expert 2, respectively (*p* = 0.47 and *p* = 0.87). Diagnostic performance metrics are illustrated in Fig. 2.

## Discussion

Our findings demonstrate poor inter-rater agreement between emergency physicians and experts regarding the quality assessment of POCUS loops performed during OHCA. While expert-to-expert agreement was substantial for the identification of cardiac standstill, agreement between operators and experts was low. Importantly, this discrepancy did not significantly affect the ability of POCUS to predict the absence of ROSC.

To our knowledge, this is the first study to evaluate inter-rater reliability between operators and experts in the specific context of OHCA. Prior literature has highlighted the need to assess the reproductibility of POCUS interpretation under real resuscitation conditions.

The study population included in this ancillary study was comparable to the overall population of the ACE cohort, supporting the external validity of the findings. Furthermore, the ACE study was multicenter, prospective and focused on early ultrasound acquisition during OHCA. The independent review of ultrasound loops by two blinded experts, who were not involved in patient inclusion or data collection, strengthen the internal validity of our analysis.

It seems that operators tend to overestimate ultrasound image quality when compared to experts’ assessments. No strong linear relationship was observed between operator and expert ratings, reinforcing the subjective nature of loop quality evaluation. While inter-expert agreement was good (high kappa), the agreement between operators and experts in identifying the presence or absence of cardiac motion is not (very low kappa).

A gap in the literature remains the non-consensual definition of cardiac motion. Isolated valvular motion can be more difficult to detect than global standstill. Furthermore, we do not have sufficient data to identify a possible link between the etiology of cardiac arrest and the patient's echogenicity to identify a potential cardiac standstill.

To our knowledge, there are no data in the literature comparing expert analysis to operator’s analysis during cardiac arrest, whether for etiological research or for the definition of cardiac standstill. However, similar investigations in other settings have studied agreement between POCUS experts and operators, particularly in the field of echocardiography. We can notably mention the meta-analysis by Albaroudi et al, which focuses on the evaluation of left ventricular systolic function (LVSF) and concludes that there is substantial agreement between emergency physicians and experts in their evaluation of LVSF.^9^ This suggests that the reliability of POCUS may vary depending on the parameter assessed and the clinical context. It seems necessary to identify the indications where clinical ultrasound performed by the emergency physician requires greater training or experience. Some studies evaluate the learning curve of each parameter in ultrasound training.^10^ The learning curve for assessing the presence or absence of cardiac motion during out-of-hospital cardiac arrest has not been evaluated to our knowledge, and would be hampered in particular by the lack of consensus on the definition of cardiac standstill. These clinicians, who are not experts in transthoracic imaging, are also faced with a difficult environment: on the ground, with a rapid reading during CPR interruption for a limited time, and using an ultra-portable device that is not always as efficient as a non-portable device.^11^, ^12^

Despite these limitations, the diagnostic performance of POCUS in predicting ROSC (sensitivity, specificity, PPV, NPV) was similar between emergency physicians and experts. As a reminder, in the ACE study, the PPV of early POCUS cardiac standstill for the absence of ROSC was 84.0 %, 95 % CI [78.3–88.6].^6^ This may be explained by evidence suggesting that minimal cardiac motions are probably associated with poor prognosis but, the study design relied on a dichotomous criterion of the absence or presence of cardiac motion. It would therefore be interesting to investigate these findings by incorporating the quantity of motion rather than limiting the assessment to its presence or absence. Furthermore, we did not analyze the loops that showed discrepancies between experts and operators, and we can therefore only assume that these cases involved minimal cardiac motion. Indeed, Teran et al. notably demonstrated that the prognostic value of POCUS was more influenced by the quantity of motion than by its presence or absence, supporting the notion that even minimal motion should be considered unfavorable.^13^

These findings support the need for further research into the determinants of high-quality, reproductible ultrasound imaging in the OHCA settings. Identifying specific criteria for acceptable image quality, as well as defining the level of training required would be valuable. In future studies, we need to investigate what constitutes a movement “worth considering”.

To mitigate operator-dependant variability, emerging approaches – such as decentralized support to assist the operator in the interpretation of ultrasound – are being explored and may offer promising avenues for future.^14^

### Limitations

This study has several limitations:

Firstly, a substantial proportion of loops were deemed non-interpretable, which limited the number of analyzable cases and may have introduced selection bias. Secondly, we did not assess the level of experience of the operators, although it is likely that more experienced clinicians would perform better. This remains a key variable for future investigation. Thirdly, image quality ratings were based on subjective expert judgment. Standardized quality criteria – such as those proposed by Gaspari et al.^15^ – were published after the initiation of the ACE study and were not used in this analysis. Fourthly, for we maintained the dichotomous criterion (movement/no movement), which means we cannot determine which cases involved minimal cardiac motion nor whether these patients achieved ROSC. Fifthly, this study was observational, and we cannot, of course, rule out the possibility of a self-fulfilling prophecy. Finally, the impact of ventilation was not evaluated. We were unable to differentiate patients receiving mechanical ventilation versus bag-valve-mask ventilation, which may influence cardiac imaging. While the effects of ventilation on cardiac ultrasound parameters are well described in spontaneously breathing patients,^16^ they are less understood during cardiac arrest, though some studies have begun to explore optimal ventilation strategies in this context.^17^

## Conclusion

This ancillary analysis of the ACE trial highlights the limited agreement between emergency physicians and ultrasound experts in assessing POCUS quality and interpreting cardiac motion during OHCA. Despite this variability, prognostication of ROSC based on ultrasound findings remained consistent across operator groups.

Further studies are required to assess minimal contractions during ROSC rather than relying solely on a binary (movement/no movement) assessment, and interpretation is not straightforward, particularly in the out-of-hospital setting. They’re also warranted to determine the required training and to evaluate the generalizability of these findings across different prehospital care systems (which differ from the French model) and healthcare professionals.

## Use of AI tool

During the preparation of this work the authors used ChatGPT in order to correct syntax and spelling. After using this tool/service, the authors reviewed and edited the content as needed and take full responsibility for the content of the published article.

## CRediT authorship contribution statement

**Mathilde Papin:** Writing – review & editing, Writing – original draft, Visualization, Investigation. **Thibaut Markarian:** Writing – review & editing, Investigation. **Quentin Le Bastard:** Writing – review & editing, Investigation. **Christelle Volteau:** Writing – review & editing, Methodology. **Philippe Pes:** Writing – review & editing, Methodology, Investigation. **Philippe Le Conte:** Writing – review & editing, Investigation. **François Javaudin:** Writing – review & editing, Visualization, Supervision, Methodology, Investigation, Conceptualization.

## Funding

The ACE study and this ancillary one were funded by a grant from the French Ministry of Health (Program Hospitalier de Recherche Clinique Interrégional, PHRC-IR 2017 [Ministère de la Santé, Paris, France]).

## Declaration of competing interest

The authors declare the following financial interests/personal relationships which may be considered as potential competing interests: “TM received fees for teaching ultrasound to GE HealthCare customers (General Electric Company, Cincinnati, OH, USA). The other authors declare no conflicts of interest.”.
